# Spirometry, questionnaire and electronic medical record based COPD in a population survey: Comparing prevalence, level of agreement and associations with potential risk factors

**DOI:** 10.1371/journal.pone.0171494

**Published:** 2017-03-08

**Authors:** Floor Borlée, C. Joris Yzermans, Esmeralda Krop, Bernadette Aalders, Jos Rooijackers, Jan-Paul Zock, Christel E. van Dijk, Catharina B. M. Maassen, François Schellevis, Dick Heederik, Lidwien A. M. Smit

**Affiliations:** 1 Institute for Risk Assessment Sciences, IRAS, Utrecht University, Utrecht, The Netherlands; 2 Netherlands Institute for Health Services Research, NIVEL, Utrecht, The Netherlands; 3 Netherlands Expertise Centre for Occupational Respiratory Disorders, Utrecht, The Netherlands; 4 National Institute for Public Health and the Environment (RIVM), Centre for Infectious Disease Control, Bilthoven, The Netherlands; 5 Department of General Practice & Elderly Care Medicine/EMGO Institute for Health and Care Research, VU University Medical Center, Amsterdam, The Netherlands; Universite de Bretagne Occidentale, FRANCE

## Abstract

**Background:**

COPD-diagnosis is confirmed by post-bronchodilator (BD) spirometry. However, epidemiological studies often rely on pre-BD spirometry, self-reports, or medical records. This population-based study aims to determine COPD-prevalence based on four different operational definitions and their level of agreement, and to compare associations between COPD-definitions and risk factors.

**Methods:**

COPD-prevalence in 1,793 adults from the general Dutch population (aged 18–70 years) was assessed based on self-reported data, Electronic Medical Records (EMR), and post-BD spirometry: using the FEV1/FVC below the lower limit of normal (LLN) and GOLD fixed cut-off (FEV1/FVC <0.70). Using spirometry as a reference, sensitivity was calculated for self-reported and EMR-based COPD. Associations between COPD and known risk factors were assessed with logistic regression. Data were collected as part of the cross-sectional VGO study (Livestock Farming and Neighboring Residents’ Health Study).

**Results:**

The highest prevalence was found based on spirometry (GOLD: 10.9%, LLN: 5.9%), followed by self-report (4.6%) and EMR (2.9%). Self-reported or EMR-based COPD identified less than 30% of all COPD-cases based on spirometry. The direction of association between known risk factors and COPD was similar across the four definitions, however, magnitude and significance varied. Especially indicators of allergy were more strongly associated with self-reported COPD compared to the other definitions.

**Conclusions:**

COPD-prevalence varied depending on the used definition. A substantial number of subjects with spirometry-based COPD cannot be identified with questionnaires or medical records which can cause underestimation of COPD-prevalence. The influence of the different COPD-definitions on associations with known risk factors was limited.

## Introduction

Chronic obstructive pulmonary disease (COPD) is a leading cause of mortality and morbidity worldwide and expected to increase in the coming decades [1]. Epidemiological studies estimating COPD prevalence show remarkable variation due to differences in measurement methodology [2]. Halbert et al. conducted a meta-analysis to quantify the global prevalence of COPD [2]. Objective definitions based on spirometry tended to produce higher prevalence estimates than patient reported diagnosis and physician diagnosis (9.2% versus 4.9% versus 5.2%, respectively). This likely reflects the underestimation and under-diagnosis of the disease prevalence [3]. COPD based on post-bronchodilator (BD) spirometry is therefore preferred in epidemiological studies and very common. Objective measurements are also preferred because they are not influenced by symptom-perception, recall-bias and access to health care [4]. However, the advantage of self-reports or medical records are the relatively low costs, allowing large sample sizes and “big data” analysis.

Studies comparing COPD-prevalence based on different data sources in the same population also found that the definitions used to assess COPD greatly influence prevalence estimates [5–10]. A study from de Marco et al. showed that the effect of risk factors for the development of COPD, such as gender, age and Body Mass Index (BMI), may also depend on the definition used [11]. However, most of these studies were conducted in patient populations [7,9,10]. In the few studies that compared COPD-definitions in the general population, only pre-BD spirometry results were available [5,6,11]. To our knowledge, this is the first population-based study that compares post-BD spirometry-based COPD with COPD-prevalence based on other data sources.

For spirometry-based COPD, the recommended cut-off for the Forced Expiratory Volume in 1 second (FEV1)/ Forced Vital Capacity (FVC) is the lower limit of normal (LLN) based on the Global Lung Initiative-2012 (GLI) reference equations that take into account sex, age, and height [12,13]. Another commonly used cut-off point for COPD is the ratio between post-BD FEV1 and FVC <0.70 (Global Initiative for Chronic Obstructive Lung Disease (GOLD)) [1]. This GOLD-definition has been criticized since the FEV1/FVC ratio generally decreases with age which results in over-diagnosis in elderly and under-diagnosis in younger people [14,15].

A comparison of different definitions for determining COPD-prevalence will give more insight into the possible effects of using various COPD-definitions on prevalence estimates and their associations with potential risk factors.

The objectives of this study are: 1) to compare COPD-prevalence and the level of agreement based on four different operational definitions: self-reported COPD, COPD based on general practitioners’ (GP) Electronic Medical Records (EMR) and COPD based on post-BD spirometry: LLN and GOLD-definition, 2) to compare associations between COPD (four operational definitions) and potential risk factors and severity measures and 3) to analyze COPD-prevalence based on pre-BD spirometry and to assess whether associations with potential risk factors are different from COPD based on post-BD spirometry.

## Materials and methods

### Study population

**Fig 1:**
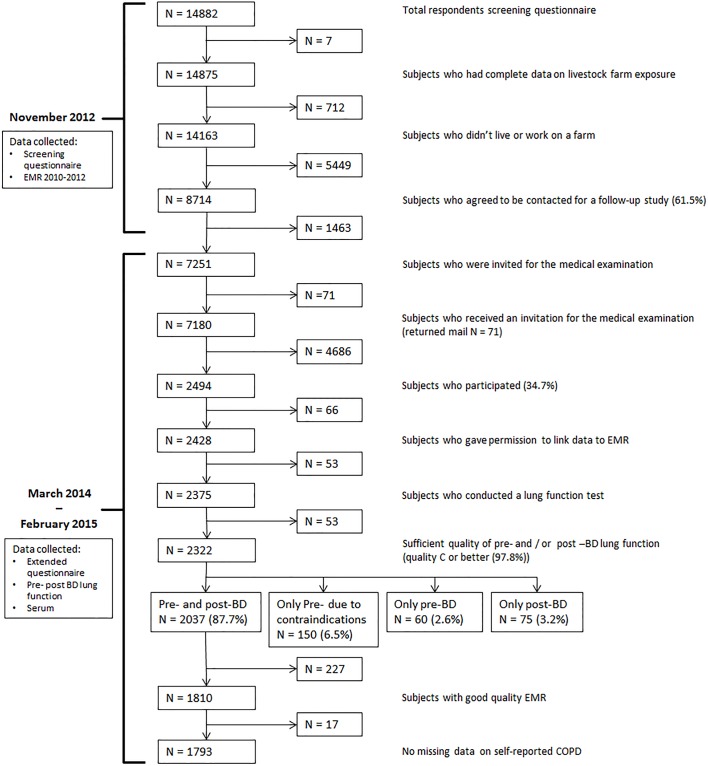
Flow chart of the data collection. Analyses are conducted on subjects with a pre-and post-bronchodilator (BD) measurement with a sufficient quality, with Electronic Medical Records (EMR) of good quality and with non-missing self-reported COPD.

Data of the present study are derived from the cross-sectional VGO study (Dutch acronym for Livestock Farming and Neighboring Residents’ Health), which aims to investigate health of residents living in the vicinity of livestock farms. In 2012, a questionnaire survey was conducted among 14,163 adults from the general population (aged 18–70 years) in the south of the Netherlands. Recruitment and inclusion criteria have been described previously by Borlée et al.[16]. Questionnaire participants who gave consent for further contact for a follow-up study, and who were not working or living on a farm were eligible for a medical survey (n = 8,714). Based on their home addresses, twelve temporary research centers were established. Between March 2014 and February 2015, all participants living within a distance of approximately 10 km of a temporary research center (n = 7,180) were invited to the nearest research center for medical examination which resulted in 2,494 participants (response 34.7%). The medical examination consisted of a second and more extended questionnaire, length and weight measurements, a lung function measurement (pre- and post-BD spirometry), collection of serum, EDTA-blood, nasal and buccal cells, and a nasal swab. In addition, fecal samples were taken by the participants at home and sent to the laboratory by mail. In this study we conducted analyses on subjects with a pre- and post-BD measurement with a sufficient quality (quality C or better), with good quality EMR available and with non-missing self-reported COPD (n = 1,793) (see Fig 1 for a flow chart of the study population).

### Ethical aspects

The VGO study protocol was approved by the Medical Ethical Committee of the University Medical Centre Utrecht (protocol number 13/533). All 2,494 subjects signed informed consent. Patients’ privacy was ensured by keeping medical information and address records separated at all times by using a Trusted Third Party.

### Data sources and COPD-definitions

#### Self-reported data

Self-reported COPD was defined as a positive answer to the question: ‘Have you ever been told by a doctor that you had chronic obstructive pulmonary disease or emphysema?’ Questionnaire data on respiratory health was assessed with the first questionnaire collected in November 2012 as described previously [16]. This was a two-page questionnaire with questions on respiratory health adopted from the European Community Respiratory Health Survey-III (ECRHS-III) screening questionnaire [17].

#### Electronic Medical Records

EMR-based COPD was defined as: ICPC-code R91 (Chronic bronchitis) or R95 (Emphysema/COPD) recorded in 2010–2012. EMR data were available through the GPs who all participated in the Netherlands Institute for Health Services Research (NIVEL) Primary Care Database [18]. The practice had to meet the following EMR quality criteria: 1) record diagnostic information in the patients’ EMR using the International Classification of Primary Care ICPC (4), 2) assign ICPC-codes to at least 70% of the morbidity records, and 3) record morbidity data at least 46 weeks per year. In addition, patients had to be registered at the GP for at least three-quarters of the year 2012. All subjects included in the data analysis gave written permission to link their study data to their EMR.

#### Spirometry

Two COPD-definitions were used based on spirometry data 1) a post-BD measurement of FEV1/FVC below the LLN, and 2) a post-BD measurement of FEV1/FVC <0.70 (GOLD). LLN was calculated with GLI-reference values based on age, gender and height [13]. Pre- and post-BD spirometry was conducted according to European Respiratory Society (ERS) guidelines and the European Community Respiratory Health Survey III (ECRHS-III) [19]. Participants stopped using inhalers and oral lung medication 4 and 8 hours prior to the lung function test, respectively. An EasyOne Spirometer (NDD Medical Technologies, Inc.) was used which measures flow and volume by ultra-sound transit time. After the pre-BD test, four puffs of short-acting beta-agonist (salbutamol, 100 μg per puff) were administered to the participant using a standard spacer. The post-BD measurement was performed at least 15 minutes after the lastly administered puff. To increase the quality of the spirometry data, we attempted to obtain four acceptable spirograms (pre- and post) per subject. The quality of all lung function curves were manually reviewed in NDD software by a specialist. The three best curves were selected or ranked manually when the best curves that were chosen by the NDD software program were not the best curves based on predefined ATS/ERS and NDD criteria [20]. In total 97.8% of the participants who conducted a lung function test had a pre- and/or post-BD measurement with a quality of C or higher (quality C: at least two reproducible curves or reproducibility within 200 ml) (N = 2,322/2,375, respectively see Fig 1).

### Potential risk factors and severity measures of COPD

Patient characteristics and severity measures of COPD were collected with the second, more extended, questionnaire which subjects completed before the medical examination. The questionnaire comprised amongst others items on symptoms and diseases, smoking habits, education and profession. Body Mass Index (BMI, kg/m^2^) was assessed during the medical examination. Atopy was defined as the presence of specific serum IgE antibodies to one or more common allergens and/or a total IgE higher than 100 IU/ml. Specific IgE to common allergens (house dust mite, grass, cat and dog) and total IgE levels were determined in serum with enzyme immunoassays as described before [21]. To gain more insight into asthma-COPD overlap, we included self-reported current asthma as a potential risk factor. Self-reported current asthma was defined as: self-reported ever asthma AND either one or more asthma-like symptoms (wheezing/whistling in the chest, chest tightness, shortness of breath at rest/following strenuous activity/at night-time or asthma attacks) or use of inhaled or oral medication for breathing problems in the last year (described before by de Marco et al.[22]. Three severity measures for COPD were computed for all participants: GOLD grades [1], self-reported health status, and the Clinical COPD Questionnaire (CCQ)-score [23]. The CCQ-score is developed to identify activity limitations and emotional dysfunction of COPD patients.

### Statistical analysis

We conducted a detailed non-response analysis in order to detect possible selection bias. Characteristics of different population subsets were compared (see Fig 1 and Table 1). The likelihood of agreeing to follow-up, or being a participant was modeled for different characteristics with logistic regression and adjusted for age, gender and smoking habits. In order to study the effect of potential selection bias, we compared the association between self-reported COPD and risk factors among different populations subsets (see S1 Table.).

**Table 1:** Comparison of characteristics of subjects who agreed and disagreed to be contacted for a follow-up study, and subjects who participated and did not participate to the medical examination.

	Agreed to follow-up	Disagreed to follow-up	Adjusted OR (95% CI)	Participated	Invited, but did not participate	Adjusted OR (95% CI)
Subjects n	8714	5449		2494	4686	
Age, mean years (SD)*	51.1 ± 12.9	49.8 ± 13.9	**1.07 (1.04–1.10)**	54.7 ± 11.0	49.1 ± 13.3	**1.49 (1.43–1.56)**
Female	53.0	54.7	0.94 (0.87–1.00)	54.6	52.2	**1.20 (1.08–1.32)**
Never smoker	45.5	49.1	1	45.0	46.4	1
Former smoker	38.8	31.4	**1.28 (1.18–1.38)**	44.6	35.7	**1.20 (1.08–1.33)**
Current smoker	15.4	17.4	0.94 (0.85–1.04)	10.1	17.7	0.97 (0.86–1.08)
**Self-reported morbidity**						
Current asthma	5.9	4.3	**1.46 (1.24–1.71)**	4.9	5.9	0.94 (0.75–1.18)
COPD	4.7	4.0	1.14 (0.96–1.35)	5.1	4.3	1.03 (0.81–1.30)
**Morbidity based on EMR**						
Subjects included with good quality EMR data n	6689	4253		1906	3359	
Asthma (ICPC R96)%	7.2	6.2	**1.19 (1.02–1.39)**	5.9	7.0	0.87 (0.68–1.11)
COPD (ICPC R95 or R091)	3.7	3.3	1.04 (0.84–1.29)	3.7	3.5	0.82 (0.60–1.11)

Data are presented as mean ±SD or %, unless otherwise stated. The likelihood of agreeing to follow-up / being a participant is modeled for different characteristics with logistic regression. OR (95% CI) were adjusted for age, gender and smoking habits. Bold type indicates statistical significance (p < 0.05). ICPC: International Classification of Primary Care.

*OR(95% CI) for an increase per 10 year.

Agreement between the presence of COPD based on the three different data sources was determined by calculating Cohen’s kappa. Using the results of the post-BD spirometry as reference standards for COPD, sensitivity, specificity, positive predictive value (PPV) and negative predictive value (NPV) for self-reported and EMR-reported COPD were calculated.

The association between each potential risk factor or severity measure with COPD was assessed by means of multiple logistic regression analysis. All analyses were adjusted for age (as a continuous variable), gender and smoking habits. To include both the qualitative effect of smoking status and the quantitative effect of smoking exposure, we included ever smoking and pack-years of smoking together as confounders [24]. Sensitivity analyses were conducted: 1) with COPD based on pre-BD measurements; 2) on subjects aged ≥40 years, since COPD diagnosis is more reliable in older patients [25,26]; and 3) after excluding subjects with self-reported current asthma.

Data were analyzed using SAS 9.4 (SAS Institute Inc., Cary, NC, USA).

## Results

### Non-response analysis

Subjects who agreed to be contacted for a follow-up study were slightly older (mean age 51.1 *vs*. 49.8 years), were more often former smokers (38.8% *vs*. 31.4%) and had more often asthma (both self-reported and EMR-based asthma) compared to subjects who disagreed (Table 1). Subjects who participated in the medical examination were older (mean age 54.7 *vs*. 49.1 years), more often female (54.6% *vs*. 52.2%) and more often former smokers (44.6% *vs*. 35.7%) compared to subjects who were invited but did not participate. Selection bias did not seem to affect associations between potential risk factors and self-reported COPD in different population subsets (S1 Table).

### COPD-prevalence and the level of agreement

The highest COPD-prevalence was found based on spirometry using the GOLD-definition (10.9%), followed by LLN-definition (5.9%), self-report (4.6%) and EMR (2.9%) (see Table 2).

**Table 2:** Prevalence and lung function characteristics for four different definitions of COPD, based on three sources: self-reported data, GP Electronic Medical Records, and spirometry.

	Self-reported questionnaire	Electronic Medical Records	Spirometry LLN	Spirometry GOLD
**COPD-definition**	A positive answer to the following question: Have you ever been told by a doctor that you had chronic obstructive pulmonary disease or emphysema?	ICPC R91(Chronic bronchitis) or R95 (Emphysema/COPD) found in years 2010–2012	A post-bronchodilator measurement of FEV1/FVC lower than lower limit of normal	A post-bronchodilator measurement of FEV1/FVC < 0.70
N (%)	82 (4.6%)	52 (2.9%)	105 (5.9%)	196 (10.9%)
Age	61.7 ± 8.7	63.4 ± 7.2	59.5 ± 9.3	62.1 ± 7.2
Female, n (%)	35 (42.7%)	26 (50.0%)	44 (41.9%)	67 (34.2%)
Never smoker, n (%)	25 (30.5%)	7 (13.5%)	16 (15.3%)	33 (16.8%)
Former smoker, n (%)	44 (53.7%)	32 (61.5%)	51 (48.6%)	104 (53.1%)
Current smoker, n (%)	13 (15.9%)	13 (25.0%)	38 (36.2%)	59 (30.1%)
**Lung function pre-measurement**				
FEV1% predicted	79.5 ± 22.5	69.9 ± 21.8	73.6 ± 18.8	81.1 ± 18.2
FVC % predicted	96.0 ± 15.7	91.6 ± 15.8	98.3 ± 18.2	101.1 ± 16.5
FEV1/FVC % predicted	81.8 ± 16.3	75.3 ± 16.8	74.0 ± 10.8	79.4 ± 10.1
MMEF % predicted	58.6 ± 35.9	44.6 ± 29.7	38.1 ± 16.1	48.3 ± 19.0
**Lung function post-measurement**				
FEV1% predicted	83.9 ± 21.7	74.1 ± 21.1	77.9 ± 17.8	84.9 ± 17.1
FVC % predicted	98.1 ± 15.0	94.1 ± 15.6	101.2 ± 17.0	103.3 ± 15.6
FEV1/FVC % predicted	84.8 ± 16.6	78.1 ± 17.2	76.4 ± 10.3	81.5 ± 9.5
MMEF % predicted	66.4 ± 38.8	49.5 ± 31.7	42.4 ± 15.8	52.1 ± 17.6

Data are presented as mean ±SD, unless otherwise stated. Pre- and post-bronchodilator lung function variables are presented as % predicted values compared with GLI-2012 reference [13] values based on age, gender and height. In total, 1793 subjects were included who had a pre- and post-BD measurement with a quality of C or higher, with Electronic Medical Records (EMR) of good quality and with non-missing self-reported COPD. FEV1:Forced Expiratory Volume in 1 sec, FVC: Forced Vital Capacity, FEV1/FVC: Tiffeneau-index, MMEF: Maximum Mid-Expiratory Flow. ICPC: International Classification of Primary Care.

**Fig 2:**
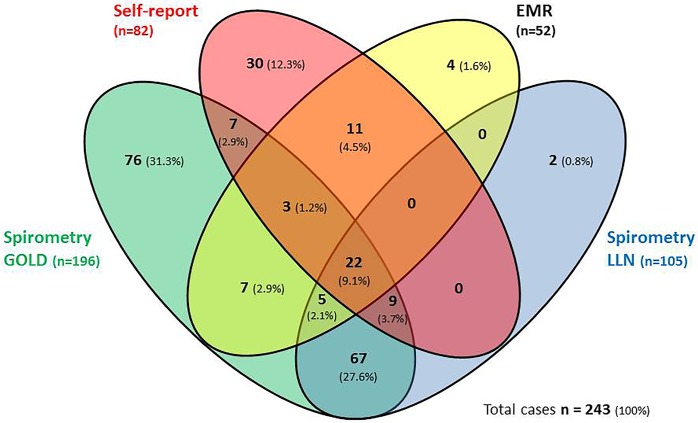
Comparison of COPD prevalence based on four different definitions, presented in n cases and in % of total identified cases. Legend Fig 2: In total 243 COPD cases were ascertained by at least one definition. In total, 1793 subjects who had a pre- and post-BD lung function measurement with a sufficient quality (C or better), Electronic Medical Records (EMR) of good quality, and without missing data on self-reported COPD (see Fig 1) were included. Self-report = self-reported data based on the ECRHSIII screening questionnaire, EMR = Electronic Medical Records, spirometry LLN = post-bronchodilator measurement of FEV1/FVC lower than FEV1/FVC-lower limits of normal, spirometry GOLD = post-bronchodilator measurement of FEV1/FVC < 0.70.

In total 243 COPD cases were ascertained by at least one definition. The overlap between COPD-prevalence based on the four different definitions was low (see Fig 2). Only 9.1% of COPD cases (n = 22) had COPD according to all four definitions. A substantial part (59.7%) was only ascertained by spirometry: 27.6% by both the LLN and the GOLD-definition, 31.3% by the GOLD-definition alone and 0.8% by the LLN-definition alone. In total, 73.2% (145/198) of spirometry-based COPD was not identified by self-reported and or EMR-based data.

The highest agreement, expressed as Cohen’s kappa [27], was found between COPD based on the two spirometry definitions (ĸ = 0.65), followed by a moderate agreement between self-reported and EMR-based COPD (ĸ = 0.52) (Table 3). Agreement between spirometry-based COPD compared with self-reported or EMR-based COPD was fair (LLN-definition: self-report: ĸ = 0.30, EMR: ĸ = 0.31, GOLD-definition: self-report: ĸ = 0.25, EMR: ĸ = 0.26).

**Table 3:** Sensitivity, specificity, positive predictive value (PPV) and negative predictive value (NPV) of COPD based on self-reported data and based on Electronic Medical Records compared with COPD based on spirometry–LLN and GOLD-definition. Agreement between the three different data sources was determined with Cohen’s Kappa.

	COPD-LLN		COPD-GOLD	
	Self-report	EMR	Self-report	EMR
**Sensitivity**	0.30	0.26	0.21	0.19
**Specificity**	0.97	0.99	0.97	0.99
**PPV**	0.38	0.52	0.50	0.71
**NPV**	0.96	0.96	0.91	0.91
**Cohen’s Kappa (95% CI)**	0.30 (0.21–0.39)	0.31 (0.22–0.41)	0.25 (0.18–0.32)	0.26 (0.19–0.34)

The agreement between GOLD-definition cases and LLN-definition cases was ĸ = 0.65 (0.60–0.72). Agreement between self-reported COPD and EMR-based COPD was (ĸ = 0.52 (0.42–0.62)). Self-report = self-reported data based on the ECRHSIII screenings questionnaire, EMR = Electronic Medical Records, COPD-LLN = COPD LLN-definition based on post-bronchodilator measurement, COPD-GOLD = COPD GOLD-definition based on post-bronchodilator measurement. The reference value was based on spirometry (LLN and GOLD). The different definitions for COPD are presented in Table 2.

Self-reported or EMR-based COPD identified less than 30% of all subjects with spirometry-based COPD (sensitivity varied between 0.19 and 0.30, specificity: 0.97–0.99) (Table 3).

As expected, since LLN is a subset of the GOLD definition, the proportion of subjects with self-reported or EMR-based COPD confirmed by spirometry-based COPD (PPV) was higher when compared with the GOLD-definition (PPV self-report: 0.50, PPV EMR: 0.71) than with the LLN-definition (PPV self-report: 0.38, PPV EMR: 0.52).

### Associations between COPD-definitions and potential risk factors and severity measures

Overall, the direction of associations was consistent across all four COPD-definitions (Table 4). A low BMI (<20 *vs*. 20–25) and pack years of smoking were significant risk factors for each COPD-definition with comparable magnitude. However, the magnitude and significance of other associations varied between the definitions.

**Table 4:** Associations of patients’ characteristics and severity measures with four different definitions of COPD.

		COPD			
	Total population	Self-report	EMR	Spirometry LLN	Spirometry GOLD
N (%)	1793 (100%)	82 (4.6%)	52 (2.9%)	105 (5.9%)	196 (10.9%)
Age (per 10 years), mean (SD)	56.2 ± 10.8	**1.72 (1.29–2.29)**	**2.38 (1.55–3.66)**	1.10 (0.87–1.39)	**1.81 (1.47–2.22)**
Female gender	56.0	0.68 (0.43–1.09)	1.20 (0.67–2.15)	0.74 (0.48–1.13)	**0.49 (0.35–0.68)**
Ever smoker	54.8	1.41 (0.85–2.32)	**4.18 (1.85–9.44)**	**4.10 (2.35–7.17)**	**3.50 (2.35–5.22)**
Pack years (per 10 years),Mean* (SD))	18.7 ± 18.4	**1.18 (1.05–1.32)**	**1.20 (1.06–1.35)**	**1.31 (1.19–1.44)**	**1.23 (1.13–1.35)**
Occupational exposure to vapors, gases, dust or fumes	32.3	1.12 (0.68–1.86)	1.49 (0.79–2.83)	1.05 (0.67–1.65)	1.17 (0.82–1.66)
BMI < 20 (ref = BMI 20–25)	1.8	**7.27 (2.33–22.68)**	**9.57 (2.53–36.18)**	**6.44 (2.43–17.11)**	**2.91 (1.04–8.11)**
BMI > 25 (ref = BMI 20–25)	65.5	0.86 (0.51–1.44)	0.77 (0.41–1.46)	**0.57 (0.36–0.90)**	**0.57 (0.40–0.82)**
High education level (ref = low/ medium)	29.5	0.63 (0.35–1.13)	**0.26 (0.09–0.73)**	**0.47 (0.27–0.83)**	**0.63 (0.42–0.94)**
Current asthma	4.3	**32.18 (17.3–59.9)**	**7.19 (3.24–15.96)**	**2.48 (1.18–5.19)**	**2.52 (1.33–4.79)**
Self-reported ever allergy	39.9	**3.23 (1.98–5.26)**	**1.87 (1.03–3.39)**	1.22 (0.79–1.88)	1.29 (0.91–1.81)
Atopy	28.2	**2.36 (1.47–3.78)**	1.82 (0.99–3.34)	1.30 (0.83–2.04)	1.04 (0.72–1.50)
≥1 positive specific IgE	20.1	**2.63 (1.58–4.38)**	1.75 (0.86–3.55)	1.46 (0.88–2.44)	1.29 (0.85–1.96)
Total IgE > = 100 IU/ml	16.4	**2.77 (1.68–4.55)**	**2.55 (1.35–4.80)**	**1.92 (1.20–3.08)**	1.37 (0.91–2.06)
GOLD-1 † (ref = FEV1/FVC > 0.7)	7.3	**2.65 (1.26–5.54)**	**4.34 (1.62–11.60)**	**NA**	**NA**
GOLD 2–4 † (ref = FEV1/FVC > 0.7)	3.6	**28.15 (14.3–55.3)**	**53.1 (24.1–117.1)**	**NA**	**NA**
CCQ-score ‡, mean (SD)	0.55 ± 0.57	**3.95 (2.92–5.33)**	**4.01 (2.80–5.75)**	**2.97 (2.25–3.92)**	**2.15 (1.68–2.76)**
Less than good self-reported health §	20.9	**6.23 (3.81–10.18)**	**6.66 (3.58–12.41)**	**2.29 (1.48–3.53)**	**1.55 (1.09–2.23)**

Data are presented as mean ±SD or %, unless otherwise stated. OR and 95% CI were adjusted for age, gender, ever smoking and pack years (number of pack years was mean-centered for ex- and current smokers). Bold type indicates statistical significance (p <0.05). Self-reported: self-reported data based on the ECRHSIII screening questionnaire, EMR: Electronic Medical Records, spirometry: post-bronchodilator lung function measurement. Used definitions for COPD based on different databases are presented in Table 2. NA: Not available, as no (GOLD) or very few (LLN) subjects with spirometry-based COPD had FEV1/FVC > 0.7.

*Mean packyears are calculated for ex-smokers and current smokers.

† GOLD-1: FEV1/FVC<0.70 and FEV1≥ 80% predicted, GOLD-2-4: FEV1/FVC <0.7 and FEV1 <80% predicted.

‡ Clinical COPD Questionnaire (CCQ)-score [23].

§ Less than good self-reported health: bad/moderate/reasonable, reference category: good/excellent self-reported health.

In particular, the association of age and gender with COPD varied according to the definition used. Age was significantly positively associated with COPD, except when the LLN-definition was used. The negative association with female gender was only statistically significant when the GOLD-definition was used, whereas the EMR-based definition showed a non-significant positive association. The positive association between self-reported allergy and COPD was only significant when using self-reported COPD or EMR-based COPD. When focusing on indicators for objectively measured allergy, we found strong positive associations between self-reported COPD and all three definitions of IgE sensitization (>1 positive specific IgE, total IgE > = 100 IU/ml, and a combination of both). EMR-based COPD and COPD based on LLN-definition were only associated with total IgE > = 100 IU/ml. Current asthma was positively associated with all four definitions, nonetheless, a substantially stronger association was observed with self-reported COPD. Indicators for COPD severity were positively associated with COPD regardless of the definition used, but stronger associations were observed with self-reported and EMR-based COPD.

Sensitivity analyses of the 1626 subjects aged ≥40 years showed a small increase in COPD-prevalence based on all four definitions (self-report: n = 81 (5.0%), EMR: n = 52 (3.2%), LLN: n = 103 (5.7%), GOLD: n = 196 (12.1%))(S2 Table). The associations between COPD and potential risk factors did not change. Analyses without patients with current asthma showed a lower prevalence of self-reported COPD (n = 52 (3.0%) *vs*. n = 82 (4.6%)), prevalence based on the other definitions did not show major changes (S3 Table). A stronger association was observed between self-reported COPD and age and a low BMI. The association between self-reported COPD and self-reported allergy and indicators for objectively measured allergy became weaker.

### Pre- *versus* post bronchodilator spirometry

COPD-prevalence increased when using pre-BD measurements (LLN pre: 9.1% *vs*. post: 5.9%, GOLD pre: 16.4% *vs*. post: 10.9%) (see Table 5). In general, similar associations with risk factors were identified by using pre- or post-BD spirometry, although associations were stronger and more often significant when COPD was based on post-measurements.

**Table 5:** Associations between spirometry-based COPD and potential risk factors and severity measures. COPD is defined on pre-and post-measurements and on LLN-definition and GOLD-definition.

	Spirometry LLN		Spirometry GOLD	
	Pre-measurement	Post-measurement	Pre-measurement	Post-measurement
N (%)	163 (9.1%)	105 (5.9%)	294 (16.4%)	196 (10.9%)
Age (per 10 years)	0.91 (0.77–1.07)	1.10 (0.87–1.39)	**1.41 (1.21–1.64)**	**1.81 (1.47–2.22)**
Female gender	0.77 (0.55–1.08)	0.74 (0.48–1.13)	**0.45 (0.35–0.60)**	**0.49 (0.35–0.68)**
Ever smoker	**2.75 (1.87–4.06)**	**4.10 (2.35–7.17)**	**2.40 (1.79–3.23)**	**3.50 (2.35–5.22)**
Pack years (per 10 years)*	**1.25 (1.14–1.37)**	**1.31 (1.19–1.44)**	**1.21 (1.12–1.32)**	**1.23 (1.13–1.35)**
Occupational exposure to vapors, gases, dust or fumes	1.01 (0.69–1.47)	1.05 (0.67–1.65)	1.04 (0.77–1.40)	1.17 (0.82–1.66)
BMI < 20 (ref = BMI 20–25)	**3.54 (1.48–8.46)**	**6.44 (2.43–17.11)**	**4.27 (1.79–10.18)**	**2.91 (1.04–8.11)**
BMI > 25 (ref = BMI 20–25)	**0.61 (0.43–0.88)**	**0.57 (0.36–0.90)**	**0.68 (0.50–0.92)**	**0.57 (0.40–0.82)**
High education level (ref = low/ medium)	0.77 (0.52–1.14)	**0.47 (0.27–0.83)**	0.81 (0.59–1.11)	**0.63 (0.42–0.94)**
Current asthma	**2.93 (1.62–5.29)**	**2.48 (1.18–5.19)**	**2.97 (1.72–5.12)**	**2.52 (1.33–4.79)**
Self-reported ever allergy	1.15 (0.81–1.63)	1.22 (0.79–1.88)	1.07 (0.81–1.43)	1.29 (0.91–1.81)
Atopy	1.41 (0.98–2.01)	1.30 (0.83–2.04)	1.07 (0.79–1.45)	1.04 (0.72–1.50)
> 1 positive for specific IgE	1.36 (0.90–2.05)	1.46 (0.88–2.44)	1.15 (0.82–1.63)	1.29 (0.85–1.96)
Total IgE > = 100 IU/ml	**1.95 (1.33–2.87)**	**1.92 (1.20–3.08)**	**1.53 (1.09–2.15)**	1.37 (0.91–2.06)
CCQ-score †, mean (SD)	**2.10 (1.65–2.68)**	**2.97 (2.25–3.92)**	**1.76 (1.42–2.19)**	**2.15 (1.68–2.76)**
Less than good self-reported health ‡	**1.86 (1.29–2.68)**	**2.29 (1.48–3.53)**	**1.44 (1.06–1.97)**	**1.55 (1.09–2.23)**

OR and 95% CI were adjusted for age, gender, ever smoking and pack years (number of pack years was mean-centered for ex- and current smokers). Bold type indicates statistical significance (p <0.05). Spirometry: pre and post-bronchodilator lung function measurement. Used definitions for COPD based on different databases are presented in Table 2.

*Mean packyears are calculated for ex-smokers and current smokers.

† Clinical COPD Questionnaire (CCQ)-score [23].

‡Less than good self-reported health: bad/moderate/reasonable, reference category: good/excellent self-reported health.

## Discussion

In a general population sample of adults aged 20–72 years from the Netherlands, we found that COPD-prevalence varied depending on the used definition (2.9–10.9%). The overlap between COPD-prevalence based on the four different operational definitions was low. Self-reported or EMR-based COPD identified less than 30% of all subjects with spirometry-based COPD, but specificity was high. Despite the variation in prevalence estimates, low overlap and low sensitivity, the direction of associations between potential risk factors and all four operational definitions of COPD were more or less similar, although the magnitude and statistical significance of the associations varied between the definitions. The combination of a relatively low prevalence and high specificity of self-reported and EMR-based COPD compared to both LLN and GOLD as a reference explains the minor changes in the associations between risk factors with the different COPD-definitions [28]. A high specificity causes a relatively low number of ‘false positive’ COPD cases in the ‘true positive’ COPD group. COPD-prevalence was substantially higher based on pre- instead of post-BD measurements. We found similar associations with risk factors when using pre- or post-BD spirometry, but the associations with risk factors were stronger and more often significant when COPD was based on post-BD measurements.

The highest COPD-prevalence was found based on the GOLD-definition (10.9%), followed by spirometry LLN-definition (5.9%), self-report (4.6%) and EMR (2.9%). Prevalence estimates were comparable with spirometry-based prevalence estimates in the larger general population studies. The PLATINO-study (n = 5,571, age ≥ 40 years) in Latin America found a prevalence varying between 7.8%-19.7% based on post-BD GOLD-definition [26]. The BOLD-study (n = 9,425, 52–60 years) estimated world-wide COPD-prevalence to be 10.1% (GOLD-2 or higher) based on post-BD measurements [25]. The number of studies in the general population comparing prevalence estimates based on different data sources is limited. Celli et al. found a twice as high COPD-prevalence based on pre-BD spirometry compared with self-reported COPD (self-report: 7.7%, LLN: 14.2%, GOLD: 16.8%) in the United States population (n = 9,838, mean±SD 48.3 ± 13.6 years)[6]. Despite the use of pre-BD spirometry, a Swedish study conducted in a population-based sample (n = 3,892, mean±SD 51.7 ± 10.6 years), found a lower COPD-prevalence compared with our results, (LLN: 4.2%, GOLD: 9.4%, self-report: 0.8%)[5]. This is possibly explained due to exclusion of subjects with physician-diagnosed asthma in the Swedish study. Mohangoo et al.[8](n = 12,699, mean±SD 39±23 years) found almost twice as high self-reported “asthma or COPD” prevalence compared to the prevalence based on GP data. Our study also found higher COPD-prevalence based on self-reported data compared to GP data.

Other studies also confirmed underestimation of COPD in the general population when using self-reported or EMR-data [2,3,5,7,10]. Pulmonary specialists may argue that COPD only based on spirometry is an overestimation since for a clinical COPD diagnosis also other indicators are needed like respiratory symptoms, family history of COPD, or history of exposure to risk factors [1]. We want to emphasize that this study aims to assess COPD for epidemiological usage and not for clinical case finding. Therefore COPD based on only lung function criteria is justifiable. Therefore COPD based on only lung function criteria is justifiable. On the other hand there are also arguments for underestimation of COPD prevalence based on spirometry since the likelihood of producing a reproducible spirometric measurement decreases with disease severity. We excluded non-reproducible tests and therefore it is likely to selectively exclude a higher proportion of subjects with airflow obstruction [2]. Furthermore, COPD is a slowly progressive disease and symptoms slowly worsen over time [1]. People adapt to these slowly developing respiratory problems and might be unaware of their condition and may not visit a GP. This could explain the low sensitivity of self-reported or EMR–based COPD. Furthermore, self-reported COPD or a diagnosis of COPD in EMR will also depend on the severity of the disease which is highly associated with care seeking behavior [29]. The CCQ-score and self-reported health–indicators of the severity of the disease—were both more strongly associated with self-reported- and EMR-based COPD compared to spirometry-based COPD. Decline in lung function occurs faster in earlier stages of the disease. Therefore, early diagnosis may slow disease progression by physical activity and prevention of exposure to smoke and other noxious agents. In addition, pharmacological intervention may control symptoms and improve quality of life [30,31].

A follow-up study by de Marco et al. [11] in young adults (ECRHS-II study, n = 4,636, 20–44 years old at the time of inclusion) studied risk factors of new-onset COPD and compared associations between risk factors and several pre-BD spirometric COPD-definitions. The association with LLN-based COPD incidence and gender, age, and being underweight lost their statistical significance compared to GOLD-based COPD incidence. We found similar associations with age, gender and underweight and these associations were also stronger with pre-BD GOLD-based COPD compared with LLN. However, being underweight was stronger associated with LLN-based COPD than GOLD when using post-BD measurements.

In our study, most associations between risk factors and different COPD-definitions had a similar magnitude and overlapping confidence intervals, except for the associations with allergy indicators. We found strong positive associations between self-reported COPD and indicators for allergy. Allergy is associated with asthma, and the association between COPD and current asthma was more prominent for self-reported COPD than for the other COPD-definitions. The associations between self-reported COPD and allergy indicators became weaker when subjects with current asthma were excluded, this indicates that some misclassification in self-reported COPD may be present due to overlap with asthma. Except for allergy indicators, this study overall indicates that for epidemiological studies with the aim to evaluate risk factors for COPD, the influence of the used definition seems to be limited. However, we focused only on risk factors that are known to be associated with COPD, and we can only speculate that the influence of different COPD-definitions on associations with unknown risk factors is limited.

Our population-based study is unique in the simultaneous use of three different data sources to assess COPD: post-BD spirometry, GP registrations, and ECRHS questionnaire items. We applied stringent quality standards to both spirometry and EMR data. In most population-based epidemiological studies based on spirometry, only pre-BD lung function measurements are available. It is not unsurprising that the prevalence estimates were higher when COPD was based on pre-BD spirometry. By using post-BD spirometry we studied fixed airway obstruction, which will reduce the number of false-positives due to overlap with asthma [32,33]. Nevertheless, the influence of using pre-BD instead of post-BD definitions on associations with potential risk factors, including current asthma, was limited. As expected, associations were somewhat stronger and more often significant when COPD was based on post-measurements, since a reduction in the number of false-positives will reduce measurement error and consequently will strengthen risk factor associations. To the best of our knowledge, this was not studied before in a population–based study. Another strength of our study was the extensive non-response analysis from the source population up to the current study population. We previously compared characteristics of non-responders and responders of the questionnaire survey (source-population)[16]. This study continued the non-response analysis by comparing characteristics of responders and non-responders in different stages of the data collection. Participants of the medical examination were older, more often female and more often former smokers compared to subjects who were invited but did not participate. Both in the previous analysis [16] and in the present study we were able to demonstrate that selection bias did not affect the associations under study, e.g. the association between self-reported COPD and potential risk factors (see S1 Table).

The three different data sources were not collected at the same time, which is a limitation of our study. Questionnaire data on COPD were collected in November 2012, EMR from 2010–2012 were used, and spirometry was conducted between March 2014 and February 2015. However, it is unlikely that the lack of overlap is to a large degree explained by COPD development during the relatively short data collection period. Previous studies that did collect self-reported data and spirometry data synchronically, also found a large degree of non-overlap [5,6].

General population studies are often conducted in urban populations. Our study population lived in a rural area outside the larger cities and farmers were excluded. The prevalence of GP-diagnosed COPD in the study area did not differ from the prevalence in other Dutch rural areas without livestock farming (42.6 *vs*. 47.1 prevalence per 1000 for patients aged >40 years, average over 2007–2013)[34], and we have no reason to expect that agreement between different COPD-definitions would be different in other areas.

## Conclusions

The operational definition used for COPD greatly influences prevalence estimates. Self-reported or EMR-based COPD identified less than 30% of all subjects with COPD based on persistent airflow limitation, which implies that a substantial number of subjects with COPD cannot be identified by questionnaires or medical records. However, the effect of the different COPD-definitions on associations with potential risk factors was limited, except for indicators of allergy, which were more strongly associated with self-reported COPD compared to the other definitions. In addition, the use of pre-BD spirometry instead of post-BD spirometry resulted in higher prevalence estimates, but had a minimal effect on associations with potential risk factors. Researchers using these operational definitions to group individuals according to COPD status, need to be aware of the impact of such choices. Results of this study may be informative for population-based epidemiological studies with the aim to evaluate potential risk factors for COPD.

## Supporting information

S1 Table Association between self-reported COPD and several characteristics compared between different population subsets to study potential selection bias. Legend Table S1. OR and 95% CI were adjusted for age, gender, and ever smoking. Bold type indicates statistical significance (p <0.05). Self-reported COPD was defined as a positive answer to the question: ‘Have you ever been told by a doctor that you had chronic obstructive pulmonary disease or emphysema?’. The sub-populations are represented in Fig 1 (main article).
(DOCX)

S2 Table Associations between risk factors and severity measures with four different definitions of COPD, only subjects older than 39 years of age are included. Legend Table S2. Data are presented as mean ±SD or %, unless otherwise stated. OR and 95% CI were adjusted for age, gender, ever smoking and pack years (number of pack years was mean-centered for ex- and current smokers). Bold type indicates statistical significance (p <0.05). Self-reported: self-reported data based on the ECRHSIII screening questionnaire, EMR: Electronic Medical Records, spirometry: post-bronchodilator lung function measurement. Used definitions for COPD based on different databases are presented in Table 2. *Mean packyears are calculated for ex-smokers and current smokers. † GOLD 1: FEV1/FVC<0.70 and FEV1≥ 80% predicted, GOLD 2–4: FEV1/FVC <0.7 and FEV1 <80% predicted ‡ Clinical COPD Questionnaire (CCQ)-score (van der Molen et al. ‘Development, validity and responsiveness of the Clinical COPD Questionnaire.’ Health Qual Life Outcomes 2003;1:13.) NA: Not available, as very few (LLN) or no (GOLD) subjects with spirometry-based COPD had FEV1/FVC > 0.7. §Less than good self-reported health: bad/moderate/reasonable, reference category: good/excellent self-reported health
(DOCX)

S3 Table Associations between risk factors and severity measures with four different definitions of COPD, subjects with current asthma are excluded. Legend S3 Table. Data are presented as mean ±SD or %, unless otherwise stated. OR and 95% CI were adjusted for age, gender, ever smoking and pack years (number of pack years was mean-centered for ex- and current smokers). Bold type indicates statistical significance (p <0.05). Self-reported: self-reported data based on the ECRHSIII screening questionnaire, EMR: Electronic Medical Records, spirometry: post-bronchodilator lung function measurement. Used definitions for COPD based on different databases are presented in Table 2. *Mean packyears are calculated for ex-smokers and current smokers. † GOLD 1: FEV1/FVC<0.70 and FEV1≥ 80% predicted, GOLD 2–4: FEV1/FVC <0.7 and FEV1 <80% predicted ‡ Clinical COPD Questionnaire (CCQ)-score (van der Molen et al. ‘Development, validity and responsiveness of the Clinical COPD Questionnaire.’ Health Qual Life Outcomes 2003;1:13.) NA: Not available, as very few (LLN) or no (GOLD) subjects with spirometry-based COPD had FEV1/FVC > 0.7. §Less than good self-reported health: bad/moderate/reasonable, reference category: good/excellent self-reported health.
(DOCX)
