# Supplementary material for: Spirometry, questionnaire and electronic medical record based COPD in a population survey: Comparing prevalence, level of agreement and associations with potential risk factors
Source: PLoS One. 2017 Mar 8;12(3):e0171494. doi: 10.1371/journal.pone.0171494 (PMC5342260; doi:10.1371/journal.pone.0171494)
Supplement: S1 Table — Legend Table S1. OR and 95% CI were adjusted for age, gender, and ever smoking. Bold type indicates statistical significance (p <0.05). Self-reported COPD was defined as a positive answer to the question: ‘Have you ever been told by a doctor that you had chronic obstructive pulmonary disease or emphysema?’. The sub-populations are represented in Fig 1 (main article). (DOCX) [file pone.0171494.s001.docx]

**Supporting Information**

**S1 Table. Association between self-reported COPD and several characteristics compared between different population subsets to study potential selection bias.**

|  | **Self-reported COPD** | | | |
| --- | --- | --- | --- | --- |
| **Characteristics** | **Respondents of screening questionnaire who are eligible for follow-up study** | **Subjects who agreed to be contacted for a follow-up study** | **Invited subjects for medical examination** | **Participants of medical examination** |
| Subjects n | 14163 | 8714 | 7180 | 2494 |
| Age (per 10 years) | **1.53 (1.42-1.66)** | **1.55 (1.41-1.72)** | **1.55 (1.39-1.73)** | **1.74 (1.39-2.16)** |
| Female gender | 1.04 (0.88-1.22) | 0.92 (0.75-1.13) | 0.87 (0.69-1.09) | 0.87 (0.61-1.25) |
| Ever smoker | **2.02 (1.68-2.43)** | **1.72 (1.37-2.16)** | **1.51 (1.18-1.94)** | **1.49 (1.01-2.20)** |
| Current asthma | **25.58 (20.77-31.50)** | **24.14 (18.74-31.09)** | **25.85 (19.44-34.39)** | **24.80 (15.29-40.23)** |

OR and 95% CI were adjusted for age, gender, ever smoking. Bold type indicates statistical significance (p <0.05). Self-reported COPD was defined as a positive answer to the question: ‘Have you ever been told by a doctor that you had chronic obstructive pulmonary disease or emphysema?’. The sub-populations are represented in figure 1 (main article).
