# Supplementary material for: Spirometry, questionnaire and electronic medical record based COPD in a population survey: Comparing prevalence, level of agreement and associations with potential risk factors
Source: PLoS One. 2017 Mar 8;12(3):e0171494. doi: 10.1371/journal.pone.0171494 (PMC5342260; doi:10.1371/journal.pone.0171494)
Supplement: S2 Table — Legend Table S2. Data are presented as mean ±SD or %, unless otherwise stated. OR and 95% CI were adjusted for age, gender, ever smoking and pack years (number of pack years was mean-centered for ex- and current smokers). Bold type indicates statistical significance (p <0.05). Self-reported: self-reported data based on the ECRHSIII screening questionnaire, EMR: Electronic Medical Records, spirometry: post-bronchodilator lung function measurement. Used definitions for COPD based on different databases are presented in Table 2. *Mean packyears are calculated for ex-smokers and current smokers. † GOLD 1: FEV1/FVC<0.70 and FEV1≥ 80% predicted, GOLD 2–4: FEV1/FVC <0.7 and FEV1 <80% predicted ‡ Clinical COPD Questionnaire (CCQ)-score (van der Molen et al. ‘Development, validity and responsiveness of the Clinical COPD Questionnaire.’ Health Qual Life Outcomes 2003;1:13.) NA: Not available, as very few (LLN) or no (GOLD) subjects with spirometry-based COPD had FEV1/FVC > 0.7. §Less than good self-reported health: bad/moderate/reasonable, reference category: good/excellent self-reported health (DOCX) [file pone.0171494.s002.docx]

**S2 Table.** **Associations between risk factors and severity measures with four different definitions of COPD, only subjects older than 39 years of age are included.**

|  | **Subjects aged 40 years and older (n=1626)** | | | |
| --- | --- | --- | --- | --- |
|  | **Self-report** | **EMR** | **Spirometry LLN** | **Spirometry GOLD** |
| N (%) | 81 (5.0%) | 52 (3.2%) | 103 (6.3%) | 196 (12.1%) |
| Age (per 10 years),mean (SD) | **1.70 (1.23-2.34)** | **2.26 (1.44-3.55)** | 1.02 (0.77-1.34) | **1.64 (1.31-2.06)** |
| Female gender | 0.71 (0.44-1.14) | 1.20 (0.67-2.14) | 0.75 (0.49-1.15) | **0.49 (0.35-0.68)** |
| Ever smoker | 1.34 (0.81-2.21) | **4.13 (1.83-9.34)** | **4.46 (2.48-8.02)** | **3.47 (2.33-5.17)** |
| Pack years (per 10 years). Mean* (SD)) | **1.19 (1.06-1.33)** | **1.20 (1.06-1.35)** | **1.30 (1.18-1.43)** | **1.23 (1.13-1.34)** |
| Occupational exposure to vapors, gases, dust or fumes | 1.17 (0.70-1.94) | 1.49 (0.79-2.82) | 1.09 (0.69-1.73) | 1.17 (0.82-1.67) |
| BMI < 20 (ref = BMI 20-25) | **7.08 (2.27-22.08)** | **9.47 (2.50-35.80)** | **5.28 (1.86-15.01)** | **2.79 (1.00-7.77)** |
| BMI > 25 (ref = BMI 20-25) | 0.83 (0.49-1.39) | 0.77 (0.41-1.46) | **0.58 (0.37-0.91)** | **0.57 (0.40-0.82)** |
| High education level (ref = low/ medium) | 0.66 (0.37-1.19) | **0.26 (0.09-0.73)** | **0.47 (0.26-0.83)** | **0.63 (0.42-0.94)** |
| Current asthma | **31.2 (16.7-58.4)** | **7.16 (3.23-15.88)** | **2.62 (1.24-5.56)** | **2.55(1.34-4.84)** |
| Self-reported ever allergy | **3.17 (1.94-5.17)** | **1.86 (1.03-3.38)** | 1.22 (0.78-1.89) | 1.28 (0.91-1.80) |
| Atopy | **2.34 (1.45-3.76)** | 1.83 (0.99-3.36) | 1.34 (0.85-2.12) | 1.05 (0.73-1.52) |
| > 1 positive for specific IgE | **2.59 (1.55-4.33)** | 1.76 (0.87-3.56) | 1.50 (0.89-2.52) | 1.30 (0.86-1.98) |
| Total IgE >= 100 IU/ml | **2.87 (1.74-4.73)** | **2.56 (1.36-4.81)** | **1.90 (1.17-3.08)** | 1.37 (0.91-2.06) |
| GOLD-1 † (ref = FEV1/FVC > 0.7) | **2.69 (1.28-5.63)** | **4.33 (1.62-11.56)** | NA | NA |
| GOLD 2-4 † (ref = FEV1/FVC > 0.7) | **28.5 (14.5-56.1)** | **52.5 (23.9-115.7)** | NA | NA |
| CCQ-score‡, mean (SD) | **3.95 (2.92-5.35)** | **4.02 (2.81-5.76)** | **3.10 (2.34-4.12)** | **2.17 (1.70-2.78)** |
| Less than good self-reported health § | **6.03 (3.68-9.88)** | **6.67 (3.58-12.43)** | **2.40 (1.55-3.72)** | **1.58 (1.10-2.26)** |

Data are presented as mean ±SD or %, unless otherwise stated. OR and 95% CI were adjusted for age, gender, ever smoking and pack years (number of pack years was mean-centered for ex- and current smokers). Bold type indicates statistical significance (p <0.05). Self-reported: self-reported data based on the ECRHSIII screening questionnaire, EMR: Electronic Medical Records, spirometry: post-bronchodilator lung function measurement. Used definitions for COPD based on different databases are presented in table 2.
*Mean packyears are calculated for ex-smokers and current smokers.
† GOLD 1: FEV1/FVC<0.70 and FEV1≥ 80% predicted, GOLD 2-4: FEV1/FVC <0.7 and FEV1 <80% predicted
‡ Clinical COPD Questionnaire (CCQ)-score (van der Molen *et al.* ‘Development, validity and responsiveness of the Clinical COPD Questionnaire.’ Health Qual Life Outcomes 2003;1:13.)
NA: Not available, as very few (LLN) or no (GOLD) subjects with spirometry-based COPD had FEV1/FVC > 0.7.
§Less than good self-reported health: bad/moderate/reasonable, reference category: good/excellent self-reported health
