# Supplementary material for: Spirometry, questionnaire and electronic medical record based COPD in a population survey: Comparing prevalence, level of agreement and associations with potential risk factors
Source: PLoS One. 2017 Mar 8;12(3):e0171494. doi: 10.1371/journal.pone.0171494 (PMC5342260; doi:10.1371/journal.pone.0171494)
Supplement: S3 Table — Legend S3 Table. Data are presented as mean ±SD or %, unless otherwise stated. OR and 95% CI were adjusted for age, gender, ever smoking and pack years (number of pack years was mean-centered for ex- and current smokers). Bold type indicates statistical significance (p <0.05). Self-reported: self-reported data based on the ECRHSIII screening questionnaire, EMR: Electronic Medical Records, spirometry: post-bronchodilator lung function measurement. Used definitions for COPD based on different databases are presented in Table 2. *Mean packyears are calculated for ex-smokers and current smokers. † GOLD 1: FEV1/FVC<0.70 and FEV1≥ 80% predicted, GOLD 2–4: FEV1/FVC <0.7 and FEV1 <80% predicted ‡ Clinical COPD Questionnaire (CCQ)-score (van der Molen et al. ‘Development, validity and responsiveness of the Clinical COPD Questionnaire.’ Health Qual Life Outcomes 2003;1:13.) NA: Not available, as very few (LLN) or no (GOLD) subjects with spirometry-based COPD had FEV1/FVC > 0.7. §Less than good self-reported health: bad/moderate/reasonable, reference category: good/excellent self-reported health. (DOCX) [file pone.0171494.s003.docx]

S3 Table. Associations between risk factors and severity measures with four different definitions of COPD, subjects with current asthma are excluded.

|  | **Subjects without current asthma (n=1716)** | | | |
| --- | --- | --- | --- | --- |
|  | **Self-report** | **EMR** | **Spirometry LLN** | **Spirometry GOLD** |
| N (%) | 52 (3.0%) | 42 (2.5%) | 95 (5.5%) | 180 (10.5%) |
| Age (per 10 years),mean (SD) | **2.28 (1.51-3.45)** | **2.76 (1.66-4.59)** | 1.06 (0.83-1.35) | **1.78 (1.44-2.21)** |
| Female gender | 0.82 (0.46-1.48) | 1.08 (0.56-2.06) | 0.78 (0.50-1.22) | **0.51 (0.36-0.72)** |
| Ever smoker | 1.58 (0.83-3.01) | **3.86 (1.59-9.36)** | **4.37 (2.41-7.92)** | **3.59 (2.37-5.44)** |
| Pack years (per 10 years).Mean* (SD)) | **1.19 (1.04-1.35)** | **1.18 (1.03-1.34)** | **1.32 (1.19-1.46)** | **1.22 (1.12-1.34)** |
| Occupational exposure to vapors, gases, dust or fumes | 1.11 (0.59-2.10) | 1.19 (0.59-2.40) | 0.98 (0.61-1.59) | 1.10 (0.76-1.59) |
| BMI < 20 (ref = BMI 20-25) | **9.81 (2.61-36.91)** | **7.94 (1.78-35.39)** | **5.73 (2.05-16.03)** | 2.45 (0.83-7.25) |
| BMI > 25 (ref = BMI 20-25) | 0.63 (0.34-1.18) | 0.64 (0.32-1.27) | **0.53 (0.33-0.84)** | **0.57 (0.40-0.82)** |
| High education level (ref = low/ medium) | 0.56 (0.26-1.23) | **0.24 (0.07-0.78)** | **0.49 (0.27-0.88)** | **0.60 (0.39-0.91)** |
| Self-reported ever allergy | **2.60 (1.42-4.77)** | 1.75 (0.90-3.40) | 1.20 (0.76-1.90) | 1.19 (0.83-1.71) |
| Atopy | 1.66 (0.90-3.06) | 1.39 (0.69-2.81) | 1.14 (0.70-1.85) | 0.89 (0.60-1.32) |
| > 1 positive for specific IgE | 1.28 (0.60-2.73) | 1.12 (0.46-2.74) | 1.09 (0.61-1.95) | 0.98 (0.61-1.57) |
| Total IgE >= 100 IU/ml | **2.48 (1.31-4.70)** | **2.07 (1.00-4.29)** | **1.74 (1.04-2.92)** | 1.20 (0.77-1.86) |
| GOLD-1 † (ref = FEV1/FVC > 0.7) | 2.06 (0.72-5.86) | **6.70 (2.31-19.42)** | NA | NA |
| GOLD 2-4 † (ref = FEV1/FVC > 0.7) | **44.8 (19.8-101.1)** | **76.2 (30.4-190.5)** | NA | NA |
| CCQ-score‡, mean (SD) | **3.99 (2.77-5.75)** | **3.77 (2.55-5.59)** | **2.80 (2.07-3.78)** | **2.07 (1.58-2.70)** |
| Less than good self-reported health § | **7.15 (3.82-13.36)** | **6.09 (3.09-11.98)** | **2.12 (1.34-3.36)** | **1.47 (1.01-2.15)** |

Data are presented as mean ±SD or %, unless otherwise stated. OR and 95% CI were adjusted for age, gender, ever smoking and pack years (number of pack years was mean-centered for ex- and current smokers). Bold type indicates statistical significance (p <0.05). Self-reported: self-reported data based on the ECRHSIII screening questionnaire, EMR: Electronic Medical Records, spirometry: post-bronchodilator lung function measurement. Used definitions for COPD based on different databases are presented in table 2.
*Mean packyears are calculated for ex-smokers and current smokers.
† GOLD 1: FEV1/FVC<0.70 and FEV1≥ 80% predicted, GOLD 2-4: FEV1/FVC <0.7 and FEV1 <80% predicted
‡ Clinical COPD Questionnaire (CCQ)-score (van der Molen et al. ‘Development, validity and responsiveness of the Clinical COPD Questionnaire.’ Health Qual Life Outcomes 2003;1:13.)
NA: Not available, as very few (LLN) or no (GOLD) subjects with spirometry-based COPD had FEV1/FVC > 0.7.
§Less than good self-reported health: bad/moderate/reasonable, reference category: good/excellent self-reported health
